# Avoiding false discoveries in single-cell RNA-seq by revisiting the first Alzheimer’s disease dataset

**DOI:** 10.7554/eLife.90214

**Published:** 2023-12-04

**Authors:** Alan E Murphy, Nurun Fancy, Nathan Skene

**Affiliations:** 1 UK Dementia Research Institute at Imperial College London London United Kingdom; 2 Department of Brain Sciences, Imperial College London London United Kingdom; Korea University Republic of Korea; Seoul National University Republic of Korea

**Keywords:** gene expression, Alzheimer's disease, single-cell, single-nucleus, Human

## Abstract

Mathys et al. conducted the first single-nucleus RNA-seq (snRNA-seq) study of Alzheimer’s disease (AD) (Mathys et al., 2019). With bulk RNA-seq, changes in gene expression across cell types can be lost, potentially masking the differentially expressed genes (DEGs) across different cell types. Through the use of single-cell techniques, the authors benefitted from increased resolution with the potential to uncover cell type-specific DEGs in AD for the first time. However, there were limitations in both their data processing and quality control and their differential expression analysis. Here, we correct these issues and use best-practice approaches to snRNA-seq differential expression, resulting in 549 times fewer DEGs at a false discovery rate of 0.05. Thus, this study highlights the impact of quality control and differential analysis methods on the discovery of disease-associated genes and aims to refocus the AD research field away from spuriously identified genes.

## Introduction

Mathys et al., 2019 undertook the first single-nucleus RNA-seq (snRNA-seq) study of Alzheimer’s disease (AD). The authors profiled the transcriptomes of approximately 80,000 cells from the prefrontal cortex, collected from 48 individuals – 24 of which presented with varying degrees of AD pathology. (Mathys et al., 2019) data processing and quality control (QC) strategy for their snRNA-seq data was state of the art at this time. Furthermore, the authors took extra measures in an attempt to ensure the reliability of their results. Here, we reanalyse this data as not a criticism of the study, but as an endeavour to raise awareness and provide recommendations for rigorous analysis of single-cell and single-nucleus RNA-seq data (sc/snRNA-seq) for future studies. Most importantly, we aim to ensure that the AD research field does not focus on spuriously identified genes.

## Results and discussion

**Figure 1.**
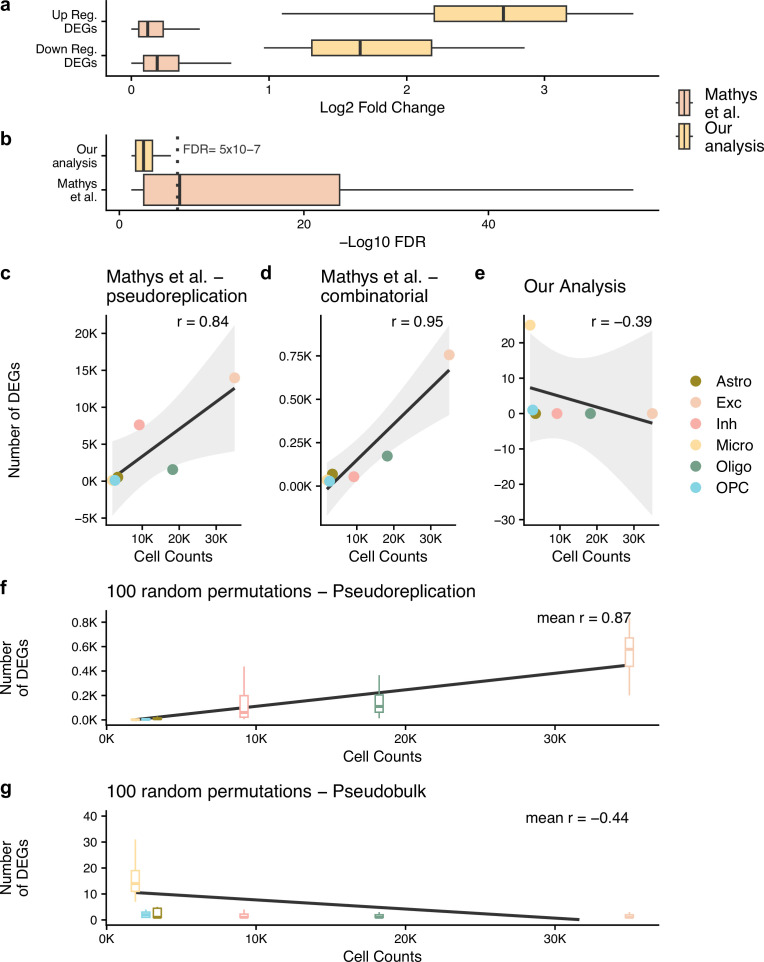
Pseudobulk differential expression results in far less dubious disease-related genes. (**a, b**) The log_2_ fold change and -log_10_ false discovery rate (FDR) of the differentially expressed genes (DEGs) from the authors’ original work (Mathys et al.) and our reanalysis (Our analysis). In (**b**), we have marked an FDR of 5 × 10^–7^, dashed grey line, to highlight how small the p-values from Mathys et al.’s analysis are. For (**a, b**), n is based on the number of DEGs: 26 for our analysis and 23,923 for Mathys et al. (**c–g**) show the Pearson correlation between the cell counts after quality control (QC) and the number of DEGs identified - n is the 6 cell types tested. For (**f, g**) analysis, the samples have been randomly mixed between case and control patients - n = 100 random permutations. The different cell types are astrocytes (Astro), excitatory neurons (Exc), inhibitory neurons (Inh), microglia (Micro), oligodendrocytes (Oligo), and oligodendrocyte precursor cells (OPC).

**Figure 2.**
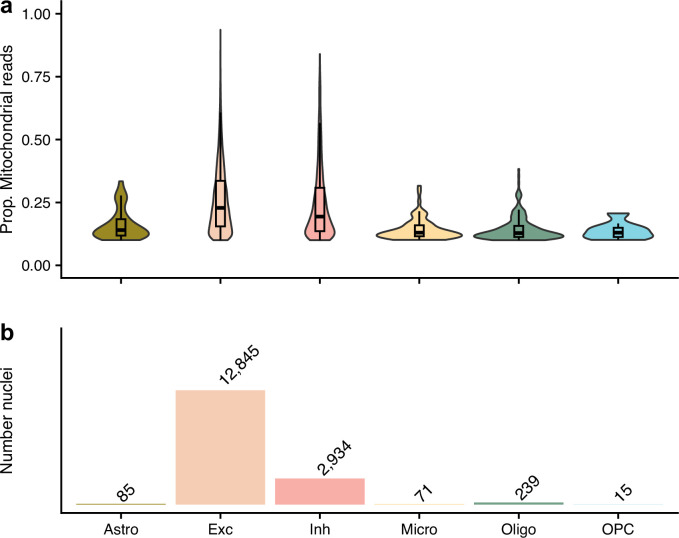
The nuclei that were removed from our quality control approach as their proportion of mitochondrial reads were ≥10%, but kept in the authors’. (**a**) shows the proportion of mitochondrial reads across the different cell types. (**b**) gives the number of removed nuclei which were kept by the authors. The different cell types are astrocytes (Ast), excitatory neurons (Ex), inhibitory neurons (In), microglia (Mic), oligodendrocytes (Oli), and oligodendrocyte precursor cells (Opc).

**Figure 3.**
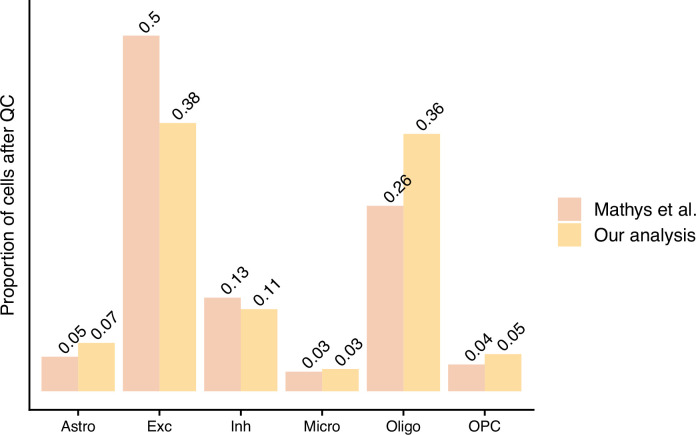
The proportion of cells left after quality control (QC) from the authors’ processing approach (Mathys et al.) and our standardised pipeline approach – scFlow (Our analysis).

Our questions of Mathys et al., 2019 focus around their data processing and their differential expression (DE) analysis (Figure 1). Firstly, in relation to their processing approach, the authors discussed the high percentages of mitochondrial reads and low number of reads per cell present in their data. This is indicative of low cell quality (Ilicic et al., 2016); however, we believe the authors’ QC approach may not capture all of these low-quality cells. Moreover, the authors did not integrate the cells from different individuals to account for batch effects. As the field has matured since the authors’ work was published, dataset integration has become a common step in sc-RNA-seq protocols and is recommended by some to remove confounding sources of variation (Heumos et al., 2023; Amezquita et al., 2020; Tran et al., 2020). To gain advantage of these recent approaches, we used scFlow (Khozoie et al., 2021) to reprocess the authors’ data. This pipeline included the removal of empty droplets, nuclei with low read counts and doublets, followed by embedding and integration of cells from separate samples and cell typing. scFlow combines best-practice approaches for processing sc/snRNA-seq datasets; see ‘Materials and methods’ for a detailed explanation of these steps. Reprocessing resulted in 50,831 cells passing QC, approximately 20,000 less than the authors’ postprocessing set with differing cell-type proportions (Figures 2 and 3).

With regards to data quality, it is worth noting that over 99% of nuclei had less than 200 genes expressed (Table 1). While this QC step was not unique to our reprocessing, the authors made the same exclusion in their analysis (Mathys et al., 2019), it highlights the relatively low quality of the data which may be attributable to the early stage of snRNA-seq technology of the time. For example, Brase et al.’s recent study of snRNA-seq of autosomal-dominant AD (Brase et al., 2023) used a more stringent cut-off for the minimum number of expressed genes and still kept 27% (122 times more) of the assayed cells after all QC steps. Moreover, the authors discussed the high percentages of mitochondrial reads in their data. The differences in approaches to filtering based on the proportion mitochondrial reads accounts for the notable discrepancy in the number of nuclei after QC between our approach and the authors’. Our approach used a 10% cut-off for the proportion of mitochondrial reads in a nuclei, as set out in Amezquita et al.’s best-practice guidelines (Amezquita et al., 2020), which is less stringent than Seurat’s guidelines (5%) (Hao et al., 2021) or that from Heumos et al., 2023 (8% from a median absolute deviations [MAD]-based cut-off selection). Conversely, the authors filtered out high mitochondrial read nuclei based on clusters from their t-SNE projection of the data (Mathys et al., 2019). Even at our lenient cut-off, over 16,000 nuclei that were removed in our QC pipeline were kept by the authors’ Figure 2, explaining the discrepancy in the number of nuclei after QC. Based on Figure 2, it is clear that the authors’ approach was ineffective at removing nuclei with high proportions of mitochondrial reads which is indicative of cell death (Heumos et al., 2023; Ilicic et al., 2016) – both excitatory and inhibitory nuclei with higher than 75% reads from the mitochondria were kept in the final processed dataset by the authors. We have made the data from our alternative processing approach publicly available (through Synapse: https://doi.org/10.7303/syn51758062.1) so that researchers can utilise this resource free of low-quality nuclei.

**Table 1.** Overview of the aggregated number of cells across samples removed at each step of the quality control (QC) as part of scFlow. Note that cells can fail QC for more than one check, so only the total failed and total passed rows will sum to 100%.

QC steps	Total cells	Percentage
Pre-QC	35,389,440	
Total failed	35,337,874	99.85
Minimum library size (n < 200)	35,307,281	99.77
Maximum library size	4742	0.01
Minimum expressed genes (n < 200)	35,312,434	99.78
Maximum library size/expressed genes (MAD> 4)	2149	0.01
Proportion of mitochondrial genes (≥ 0.1)	1,097,738	3.10
Multiplets (pK = 0.0054)	581	0.00
Total passed	51,566	0.15

MAD, median absolute deviation.

Our second question of Mathys et al., 2019 is their DE approach. The authors conducted a DE analysis between the controls and the patients with AD pathology, concentrating on six neuronal and glial cell types; excitatory neurons, inhibitory neurons, astrocytes, microglia, oligodendrocytes, and oligodendrocyte precursor cells, derived from the Allen Brain Atlas (Tasic et al., 2018). They performed downstream analysis on their identified differentially expressed genes (DEGs) and investigated some of the most compelling genes in more detail. Therefore, all findings put forward by their paper were based upon the validity of their DE approach. However, for this approach, the authors conducted a two-part, cell- and patient-level analysis. The cell-level analysis took each cell as an independent replicate, and the results of which were compared for consistency in directionality and rank of their DEGs against their patient-level analysis, a Poisson mixed model. The authors identified 1031 DEGs using this combinatorial approach – DEGs requiring a false discovery rate (FDR) < 0.01 in the cell-level and an FDR < 0.05 in the patient-level analysis. It is important to note that this cell-level DE approach, also known as pseudoreplication, overestimates the confidence in DEGs due to the statistical dependence between cells from the same patient not being considered (Murphy and Skene, 2022; Squair et al., 2021; Zimmerman et al., 2021; Lazic, 2010). When we inspect all DEGs identified at an FDR of 0.05 from the authors’ cell-level analysis, this number increases to 14,274. Pseudobulk DE analysis has recently been proven to give optimal performance compared to both mixed models and pseudoreplication approaches (Murphy and Skene, 2022; Squair et al., 2021; Crowell et al., 2020; Soneson and Robinson, 2018). It aggregates counts to individuals, thus accounting for the dependence between an individual’s cells.

Here, to compare the effect of the different DE approaches in isolation, we apply a pseudobulk DE approach (Chen et al., 2016) to the authors’ original processed data. We found 26 unique DEGs when considering the six cell types used by the authors (Table 2). This was 549 times fewer DEGs than that reported originally at an FDR of 0.05. When we compare these DEGs, we can see that the absolute log_2_ fold change (LFC) of our DEGs is 15 times larger than the authors’; median LFC of 2.34 and 0.16, despite the authors’ DEGs having an FDR score 8000 times smaller; median FDR of 2.89 × 10^–7^and 0.002 (Figure 1a and b). Although we examined a high correlation in the genes’ fold change values across our pseudobulk analysis and the authors’ pseudoreplication analysis (Pearson R of 0.87 for an adjusted p-value of 0.05, Table 3), the p-values and resulting DEGs vary considerably. The correspondence in fold change values is expected given the approaches are applied to the same dataset, whereas the probabilities, which pertain to the likelihood that a gene’s expressional changes is related to the case/control differences in AD, importantly do not align. We can show that this stark contrast is just an artefact of the authors taking cells as independent replicates and thus artificially inflating confidence by considering the Pearson correlation between the number of DEGs found and the cell counts (Figure 1c–e). There is a near perfect, positive correlation between DEG and cell counts for the authors’ pseudoreplication analysis (Figure 1c) and for the 1031 genes from the authors’ combinatorial approach (Figure 1d) which is not present in our pseudobulk reanalysis (Figure 1e).

**Table 2.** The differentially expressed genes from our reanalysis using the same processed data the authors used and pseudobulk differential expression approach.

Cell	logFC	logCPM	LR	p-Value	adj_pval	HGNC
Mic	2.70178913	6.99794619	26.1418415	3.17E-07	0.00061349	ACRBP
Mic	1.48930071	8.06240877	28.6361217	8.73E-08	0.00019303	APOC1
Mic	1.09327669	8.64199769	21.5323014	3.48E-06	0.00336416	CD81
Mic	–1.4157681	7.93884875	23.9955467	9.66E-07	0.00135806	CD83
Mic	3.3782727	6.86183548	32.0804401	1.48E-08	4.58E-05	CLEC1B
Mic	2.84072452	6.74370542	21.7745509	3.07E-06	0.00316269	EGF
Mic	2.55769658	6.78345087	18.0468872	2.16E-05	0.01699007	ELOVL7
Mic	–1.2056098	8.33197499	22.6644045	1.93E-06	0.00229576	IFI44L
Mic	–1.6616069	7.15366639	16.4801274	4.92E-05	0.03306938	IFI6
Mic	–1.9809425	7.00396289	17.9180823	2.31E-05	0.01699007	IFIT3
Mic	2.76502672	6.72978805	20.6543637	5.50E-06	0.00472825	ITGA2B
Mic	1.90963403	7.01552233	16.3200189	5.35E-05	0.03448474	MAP1A
Mic	–1.8194508	8.26208887	45.2221008	1.76E-11	1.36E-07	NAMPT
Mic	2.0945044	7.11048456	20.8068524	5.08E-06	0.00462318	NEXN
Mic	–2.3789762	6.93896985	22.3912441	2.22E-06	0.00245752	NR4A2
Mic	–2.8553462	6.73713862	22.8029868	1.79E-06	0.00229576	NR4A3
Mic	3.32873829	6.84942721	30.955327	2.64E-08	6.81E-05	PF4
Mic	3.4213986	6.87326383	33.2621657	8.05E-09	3.11E-05	PKHD1L1
Mic	3.64525677	6.93422174	38.661272	5.04E-10	2.60E-06	PPBP
Mic	2.30482679	8.10570443	60.7932697	6.34E-15	9.81E-11	PTPRG
Mic	–1.0382468	8.11450266	15.5968273	7.84E-05	0.04850839	RORA
Mic	2.54636649	6.69202981	17.2532606	3.27E-05	0.02300507	SDPR
Mic	–0.9629617	8.8434334	17.9319131	2.29E-05	0.01699007	SYTL3
Mic	–1.4215374	7.99629806	25.4736272	4.48E-07	0.00077092	TMEM2
Mic	2.98901596	6.77276641	24.2100819	8.64E-07	0.00133637	TUBB1
Opc	–2.8274718	5.03371292	22.1334581	2.54E-06	0.04176231	EGR1

CPM - Counts per Million, LR - fold change ratio, HGNC - HUGO Gene Nomenclature Committee.

**Table 3.** Pearson correlation between our pseudobulk differential expression analysis and the authors’ pseudoreplication analysis on all genes found to be significant at different adjusted p-value cut-offs from the authors’ pseudoreplication analysis.

Pseudoreplication adjusted p-value cut-off	Number of genes compared	Pearson correlation
0.01	20,152	0.8646269
0.05	23,903	0.8708275
0.1	26,382	0.8721126
0.25	32,117	0.8764692
0.5	42,022	0.8751554
1	84,467	0.826248

A further point which questions the authors’ DE approach is that they identified the vast majority of DEGs in the more abundant, neuronal cell types (Mathys et al., 2019). However, an increase in the number of cells is not the same as an increase in sample size since these cells are not independent from one another – they come from the same sample. Therefore, an increase in the number of cells should not necessarily result in an increase in the number of DEGs, whereas an increase in the number of samples would. This point is the major issue with pseudoreplication approaches which overestimate confidence when performing DE due to the statistical dependence between cells from the same patient not being considered (Squair et al., 2021; Lazic, 2010). In our opinion, it makes more sense to identify the majority of large effect size DEGs in microglia which recent work has established is the primary cell type by which the genetic risk for AD acts (Skene and Grant, 2016; McQuade and Blurton-Jones, 2019). This is what we found with our pseudobulk DE approach – 96% of all DEGs were in microglia (Table 2), whereas only 3% of the authors’ DEGs were in microglia.

Although it has been proven that pseudoreplication approaches result in false positives by artificially inflating the confidence from non-independent samples, we wanted to investigate the effect of the approach on the authors’ dataset. We ran the same cell-level analysis approach – a Wilcoxon rank-sum test and FDR multiple-testing correction – 100 times whilst randomly permuting the patient identifiers (Figure 1f). We would expect to find minimal DEGs with this approach given the random mixing of case and control patients. However, this pseudoreplication approach consistently found high numbers of DEGs, and we observe the same correlation between the number of cells and the number of DEGs as with the authors’ results. We did not observe the same pattern when running the same analysis with pseudobulk DE (Figure 1g). As a result, we conclude that integrating this pseudoreplication approach with a mixed model like the authors proposed just artificially inflates the test confidence for a random sample of the genes resulting in more false discoveries in cell types with bigger counts.

Up to this point, to compare the effect of the DE approaches in isolation, we analysed the same processed data from the authors as opposed to our reprocessed data. We also performed pseudobulk DE on our reprocessed data and found 16 unique DEGs (Table 4). It is worth noting that the fold change correlation between our two DE analyses (reprocessed data vs authors’ processed data) on the identified DEGs is only moderate (Pearson R of 0.57) and is lower than that of the correlation between pseudoreplication and pseudobulk on the same dataset (Table 3). This highlights the effect that the low quality high mitochondrial read cells have on DE analysis.

**Table 4.** The differentially expressed genes from our reanalysis using the reprocessed data and pseudobulk differential expression approach.

Cell	logFC	logCPM	LR	p-Value	adj_pval	ensembl_id	HGNC
OPC	–4.1544663	4.92100803	21.6911445	3.20E-06	0.04985906	ENSG00000166573	GALR1
Astro	–4.5845276	4.7965143	22.2367847	2.41E-06	0.037634	ENSG00000137959	IFI44L
Micro	–3.7616619	7.32875316	26.8149688	2.24E-07	0.00077905	ENSG00000077238	IL4R
Micro	–2.0681446	7.88736441	17.5929095	2.74E-05	0.0346187	ENSG00000105835	NAMPT
Micro	–1.6757556	7.58472506	19.1736829	1.19E-05	0.02076348	ENSG00000118257	NRP2
Micro	–3.1556403	6.85232653	19.2064627	1.17E-05	0.02076348	ENSG00000135363	LMO2
Micro	–3.4339265	6.9290472	19.5975589	9.56E-06	0.02076348	ENSG00000138135	CH25H
Micro	–2.8183109	6.77500676	16.907959	3.92E-05	0.04550806	ENSG00000142408	CACNG8
Micro	2.90076647	8.34560617	45.5144266	1.52E-11	2.11E-07	ENSG00000144724	PTPRG
Micro	3.25867589	6.91671013	16.5519147	4.73E-05	0.0490155	ENSG00000163106	HPGDS
Micro	–2.0290905	7.12321166	16.4746746	4.93E-05	0.0490155	ENSG00000171612	SLC25A33
Micro	–3.4657301	6.93307221	19.7883301	8.65E-06	0.02076348	ENSG00000172243	CLEC7A
Micro	–4.172807	7.16813583	34.3515807	4.60E-09	3.20E-05	ENSG00000174600	CMKLR1
Micro	–3.1984588	6.87310555	18.5335889	1.67E-05	0.0232342	ENSG00000227531	RP11-202G18.1
Micro	3.40562887	6.9381703	18.5526502	1.65E-05	0.0232342	ENSG00000228058	RP11-552D4.1
Micro	4.46073301	7.66559163	29.7716679	4.86E-08	0.00022549	ENSG00000253496	RP11-13N12.1

In conclusion, the authors’ analysis has been highly influential in the field with numerous studies undertaken based on their results, something we show has uncertain foundations. However, we would like to highlight that the use of pseudoreplication in neuroscience research is not isolated to the authors’ work; others have used this approach (Fernandes et al., 2020; Lui et al., 2021; Wakhloo et al., 2020), and their results should be similarly scrutinised. Here, we provide our processed count matrix with metadata and also the DEGs identified using an independently validated, DE approach so that other researchers can use this rich dataset free from spurious nuclei or DEGs. While the number of DEGs found here is significantly lower, much greater confidence can be had that these are AD-relevant genes. The low number of DEGs found may also cause concern given the sample size and cost of collection and sequencing of such datasets. However, the increasing number of snRNA-seq studies being conducted for AD creates the opportunity to conduct differential meta-analyses to increase power. Further work is required in the field to develop methods to conduct such analysis, integrating studies and accounting for their heterogeneity, similar to that which has been done for bulk RNA-seq (Rau et al., 2014). Some such approaches have already been made in COVID-19 research which could be leveraged for neurodegenerative disease (Garg et al., 2021).

## Materials and methods

### Processing of sc/snRNA-seq dataset

The data reprocessing was conducted with scFlow (Khozoie et al., 2021), the steps of which are discussed in the following two sections.

#### Quality control of snRNAseq data

The raw snRNA-seq data (10.7303/syn18485175) and the ROSMAP metadata (10.7303/syn3157322) were downloaded from https://www.synapse.org/ upon acquiring appropriate approval. Downstream primary analyses of gene–cell matrices were performed using our scFlow pipeline (Khozoie et al., 2021). To determine ambient RNA profile and distinguish true nuclei from empty droplets, emptyDrops was used with a lower parameter of <100 counts, an alpha cut-off of ≤0.001, and with 10,000 Monte Carlo iterations (Lun et al., 2019). This approach has been recommended as best practice in the literature (Amezquita et al., 2020). Nuclei were then filtered for ≥200 total counts and ≥200 total expressed genes, which was defined as a minimum of 2 counts in at least three cells. We excluded any nuclei with total counts or total expressed genes with more than 4 MAD defined by an adaptive thresholding method. Nuclei were excluded if the proportion of counts mapping to mitochondrial genes was more than 10%, as set out in best-practice guidelines (Amezquita et al., 2020). Doublets were identified using the DoubletFinder algorithm, with a doublets-per-thousand-cells increment of eight cells (recommended by 10X Genomics), and a pK value of 0.005 (McGinnis et al., 2019). DoubletFinder was shown to be the best overall performing method in a recent benchmark (Xi and Li, 2021). The aggregated number of cells and proportions dropped at each step is given in Table 1 while a comparison of the proportion of cells in each cell type after reprocessing compared to the authors’ processed data is given in Figure 2. All files from the scFlow run, including QC statistics, are available in the GitHub repository in the scFlow_files folder (copy archived at Murphy, 2023). This includes sample-level genes and cells’ QC numbers.

#### Integration and clustering

The linked inference of genomic experimental relationships (LIGER) package was used to calculate integrative factors across samples (Welch et al., 2019). LIGER was recently found to be one of the top performing methods for batch-effect correction (Tran et al., 2020). LIGER parameters used included k: 30; lambda: 5.0; thresh: 0.0001; max_iters: 100; knn_k: 20; min_cells: 2; quantiles: 50; nstart: 10; resolution: 1; num_genes: 3000; and centre: false. Two-dimensional embeddings of the LIGER integrated factors were calculated using the Uniform Manifold Approximation and Projection (UMAP) algorithm with the following parameters: pca_dims: 50; n_neighbours: 35; init: spectral; metric: euclidean; n_epochs: 200; learning_rate: 1; min_dist: 0.4; spread: 0.85; set_op_mix_ratio: 1; local connectivity: 1; repulsion_strength: 1; negative_sample_rate: 5; and fast_sgd: false (McInnes et al., 2020). The Leiden community detection algorithm was used to detect clusters of cells from the 2D UMAP (LIGER) embeddings; a resolution parameter of 0.001 and a k value of 50 were used (Traag et al., 2019). This approach has been noted as best practice by a recent review (Heumos et al., 2023). Automated cell typing of the detected clusters was performed as previously described using the Expression Weighted Celltype Enrichment algorithm in scFlow against a previously generated cell-type data reference from the Allen Human Brain Atlas (Hodge et al., 2019; Skene and Grant, 2016). The top five marker genes for each automatically annotated cell type were determined using Monocle 3 and validated against canonical cell-type markers (Trapnell et al., 2014).

### DE analysis

All DE analyses were conducted using pseudobulk DE approach with sum aggregation and edgeR LRT (Chen et al., 2016). Pseudobulk aggregates nuclei within a biological replicate (an individual) for each cell type, reducing the dropout issue in single-cell data and avoiding the false inflation of confidence from non-independent samples of pseudoreplication approaches (Squair et al., 2021; Murphy and Skene, 2022). The DE analysis pipeline is available at GitHub repository (copy archived at Murphy, 2023). This is a general use pipeline which can be run for any single-nucleus or single-cell transcriptomic dataset. Note that we report DEGs across AD and controls using the same processed data the authors used (Table 2) and using our reprocessed data (Table 4).

### Code availability

The DE analysis pipeline is available at GitHub repository (copy archived at Murphy, 2023). This is a general use pipeline which can be run for any single-nucleus or single-cell transcriptomic dataset. The config file containing all the parameters used and QC overview file for the scFlow run is also available in this repository.

## Data Availability

The differentially expressed genes and processed count matrix from the original study are available with their manuscript. The count matrix and metadata from our reprocessing approach are available via the AD Knowledge Portal (https://adknowledgeportal.org). The AD Knowledge Portal is a platform for accessing data, analyses, and tools generated by the Accelerating Medicines Partnership (AMP-AD) Target Discovery Program and other National Institute on Aging (NIA)-supported programs to enable open-science practices and accelerate translational learning. The data, analyses, and tools are shared early in the research cycle without a publication embargo on secondary use. Data is available for general research use according to the following requirements for data access and data attribution (https://adknowledgeportal.org/DataAccess/Instructions). For access to content described in this article, see https://doi.org/10.7303/syn51758062.1. All other relevant scripts and data for working with this dataset and supporting the key findings of this study are available within the article or from our GitHub repository (copy archived at Murphy, 2023). The following previously published dataset was used: MathysH Davila-VelderrainJ PengZ GaoF MohammadiS YoungJZ MenonM HeL AbdurrobF JiangX MartorellAJ RansohoffRM HaflerBP BennettDA KellisM TsaiLH 2019Single-cell transcriptomic analysis of Alzheimer's diseaseSynapse10.7303/syn18485175PMC686582231042697

## Additional files

MDAR checklist
